# Combining Problem‐Based Learning and Team‐Based Learning in a Longitudinal Integrated Clerkship: How Do Students Spend Their Self‐Study Time?

**DOI:** 10.1111/tct.70008

**Published:** 2024-12-25

**Authors:** Ida Sara Johnsen, Vidar Gynnild, Børge Lillebo

**Affiliations:** ^1^ Department of Circulation and Medical Imaging, Faculty of Medicine and Health Sciences Norwegian University of Science and Technology (NTNU) Trondheim Norway; ^2^ Department of Education and Lifelong Learning, Faculty of Social and Educational Sciences Norwegian University of Science and Technology (NTNU) Trondheim Norway; ^3^ Clinic of Medicine and Rehabilitation Levanger Hospital Levanger Norway

**Keywords:** active learning, instructional design, learning behaviour, longitudinal integrated clerkship, self‐study

## Abstract

Self‐study is essential for student learning, and active learning methods aim to facilitate constructive self‐study time usage. How active learning strategies actually affect student self‐study in medical education, however, remains partly unknown. The aim of this study was to examine medical students' use of self‐study time in a longitudinal integrated clerkship employing active learning strategies. Week‐long time logs were used to collect quantitative data on students' scholastic activities. Furthermore, semistructured individual interviews were conducted for in‐depth exploration of different learning activities' influence on students' self‐study behaviour. Preparation for and follow‐up work after scheduled learning activities were specifically addressed. Students spent on average 42.1 h per week on educational activities, of which self‐study comprised 23 h. Preparation for upcoming learning activities was highly prioritized by the students. Team‐based learning (TBL) generated most self‐study hours, closely followed by patient encounters, while problem‐based learning (PBL) induced least amounts of self‐study. 50% of self‐study time was done without specific relation to scheduled learning activities. Active learning methods appear to promote preparatory work, in accordance with their pedagogic theory. Type of learning activity appears to be decisive in determining the amount of self‐study students choose to undertake. Future research should address how quantity and content of different learning activities influence self‐study behaviour.

## 1 Introduction

Advances in the science of learning challenge current medical education delivery [1]. Accordingly, active learning pedagogies such as problem‐based learning (PBL), team‐based learning (TBL) and longitudinal integrated clerkships (LICs) have been implemented in medical curricula internationally [2]. Such approaches engage students in theoretical discussions and practical training and aim to promote constructive self‐study of content that traditionally has been lectured [3]. However, to our knowledge, no studies have characterized both the quantity and quality of student self‐study in a medical curriculum incorporating the above‐mentioned pedagogies. This study aimed to increase the understanding of self‐study behaviour among medical students in a learner‐centred longitudinally integrated curriculum. Knowledge about this can provide insight for curriculum planning regarding type and quantity of scheduled learning activities and time set aside for self‐study.

Research on learning and memory have pointed out certain learning strategies as particularly beneficial for mastering new knowledge and skills. These include spacing, interleaving, retrieval practice, elaboration and concrete examples [4]. Spacing, or distributed practice, refers to the act of spreading out learning sessions within the same subject over time, resulting in repeated exposure with time intervals between each session [1]. Interleaving entails switching between different subjects repeatedly, as opposed to reviewing each subject separately as in blocked formats [1, 4]. Retrieval practice denotes recalling existing knowledge from the memory, an activity typically executed during summative assessments, but which may also be implemented as part of formative testing or learning settings [4]. Grounded on these principles, various active instructional strategies have been developed, aiming to facilitate constructive use of self‐study time. PBL and TBL incorporate reasoning around real‐world clinical problems, retrieving prior knowledge and engaging in elaborative discussions [5, 6]. The educational effectiveness of these pedagogies are supported by a body of research [3, 7]. Accordingly, both PBL and TBL have been implemented in numerous medical schools all over the world. It has been argued that by combining the two approaches mutual benefits can be achieved, thus optimizing student learning [5].

LICs are learner‐centred curricular models that facilitate students' active engagement in comprehensive patient care [8]. Moreover, longitudinal integration of subjects and continuity between students, clinicians and patients are core elements that incorporate the evidence‐based learning strategies spacing and interleaving [9]. Numerous investigations imply advantageous outcomes of LICs for students, clinicians and community [10].

The mentioned learning strategies: spacing, interleaving and retrieval practice can be applied to self‐study practice itself, as well as curricular activities and organization as outlined above. In a review revising the utility of different learning techniques, distributed practice and practice testing are found to enhance student performance and are thus perceived as high utility study techniques [11]. A programme aiming to teach students about effective learning methods was shown to increase students use of, for example, interleaving and distributed practice, at the expense of highlighting and rereading. Students who received this training programme also improved their academic results [12].

How students make use of self‐study time is important for their learning. Amount of time spent on self‐study also matters for academic performance. The association between self‐study time and academic achievements in higher education has been debated [13], but studies within medical education indicate a favourable interrelationship [14, 15]. Self‐study's role in the development of independent, active learning processes is suggested as a possible explanation [15].

The pedagogical approaches applied in the investigated curriculum in this study, as well as this study as a whole, are founded on constructivist learning theory. Central to this theory is the assertion that learners construct their own knowledge by activating previous skills and experiences and adjusting preceding understandings according to the new information presented [16, 17]. Engaging students through active learning methods to incite reflection upon their existing knowledge is one way of obtaining this [18]. This reflection can occur before, during or after learning activities and can apply to both in‐class and self‐study behaviour.

### 1.1 The Present Study

The Norwegian University of Science and Technology (NTNU) introduced a new decentralized curriculum within its medical degree programme in 2018, known as NTNU Link. Its structure is based on LIC principles, and in addition to clinical patient encounters, it offers regular TBL and PBL activities. These are thematically integrated and longitudinally spread out over the semester and combined with student‐directed self‐study time. Students are usually informed about the topic of upcoming learning activities, and they are encouraged to prepare for these in advance. The curricular features of NTNU Link build on the idea that active and student‐centred learning promotes lifelong learners with the ability to attain knowledge and skills that are essential to hold for future medical doctors. This is theoretically supported by constructivist learning theory and empirically underpinned by various studies demonstrating the enhanced learning effects of testing, spacing and interleaving [1].

The purpose of this study is to explore the interplay between scheduled learning activities and use of self‐study time among medical students enrolled in a longitudinally integrated curriculum applying active learning strategies. The specific research questions are as follows: (1) How do students spend their self‐study time, with respect to content and quantity? (2) How do the scheduled learning activities TBL, PBL and patient encounters influence students' self‐study behaviour?

## 2 Methods

We conducted a mixed methods study, combining qualitative and quantitative arms. Time logs were used to register students' study activity. For in‐depth exploration of students' use of self‐study time, we performed semistructured individual interviews. Ethical approval was obtained by the Norwegian Centre for Research Data (NSD number 450319). All participants gave informed consent to participate in the study.

### 2.1 Setting

NTNU Link comprises years 3 and 4 of the six‐year undergraduate medical degree programme and accommodates up to 16 students per year. It is a decentralized curriculum, located approximately 80 km away from the main campus. NTNU Link students undertake years 1 and 2 at the main campus and reunite with their urban peers again for years 5 and 6. Enrolment is voluntary, and teaching takes place at a community hospital trust and at nearby primary healthcare services. The teaching in NTNU Link is organized in four topic‐based weeks per semester, through which students rotate in groups of 2–4. As one semester consists of 16 weeks, students rotate through each week four times. During the topic‐based weeks, clinical patient encounters, TBL‐ and PBL‐sessions are carried out, covering a broad curriculum aligned to the main campus programme. Mostly, students meet the same instructors within each topic through all rotations. As opposed to the curriculum delivered at the main campus, NTNU Link applies no learning activities in the format of traditional plenary lectures. One day each month, NTNU Link students attend learning activities at the main campus, reviewing clinical subjects that are not covered in the decentralized curriculum. Summative assessments are identical for NTNU Link and the main programme and consist of end‐of‐year (year 3) and end‐of‐semester (year 4) written and oral examinations.

### 2.2 Time Logs

Three cohorts of medical students embedded in NTNU Link in 2021–2022 were invited to fill in time logs; 25 out of 28 invited students contributed with a total of 33 week‐long time logs. These time logs were informed by similar tools utilized in previous studies [19, 20]. The logs covered 24 h a day divided into 15‐min intervals. Recording categories were ‘Instruction’, ‘Self‐study’, ‘Nonscholastic activities’ and ‘Sleep’. For the two former categories, students were instructed to specify type of activity.

Time logs were completed for week 12 (cohort 1, 3^rd^ year), week 13 (cohort 2, 4^th^ year), week 43 (cohort 1, 4^th^ year) and week 44 (cohort 3, 3^rd^ year). These midsemester weeks were chosen to avoid the examination period and to cover 1 week in each semester for both study year 3 and 4. Log data were imported into Excel, which was used to calculate descriptive statistics.

### 2.3 Interviews

All 3^rd^ year medical students enrolled in NTNU Link in the autumn of 2021 were invited to participate (cohort 1). Thus, for the interviews only one cohort of students were included of feasibility reasons. Students were contacted by email for recruitment, and nine out of 10 contacted students participated. Semistructured individual interviews were undertaken between December 2021 and February 2022. All interviews were conducted by the first author, who had no previous connections with the participants. The interviews lasted between 44 and 102 min and were directed by an interview guide (see Box 1), based on previous research and the authors' experience related to the subject.

Box 1 **Interview guide. Questions and prompts utilized during semistructured individual interviews.**

Interview guide

Warm‐up questions:

- Which study year are you currently in?
- Why did you choose NTNU Link?
- What kind of learning activity do you prefer (TBL, patient encounters or PBL)?

Topic A. How do medical students use their self‐study time?

- Can you tell me about a typical study week for you, with focus on how you use your self‐study time before, between and after scheduled learning activities?
- To what degree does problem‐based learning (PBL) affect how you use your self‐study time? In what way does PBL affect your self‐study time?
- To what degree does team‐based learning (TBL) affect how you use your self‐study time? In what way does TBL affect your self‐study time?
- To what degree do patient encounters affect how you use your self‐study time? In what way do patient encounters affect your self‐study time?
- To what degree do you prepare for upcoming learning activities? (PBL, TBL, patient encounters)
  ○ How do you prepare?
  ○ What do you wish to achieve doing this?
- To what degree do you do follow‐up work after attending learning activities? (PBL, TBL, patient encounters)
  ○ How do you do follow‐up work?
  ○ What do you wish to achieve doing this?
- What is your strategy when you intend to learn something?
- How do you work during self‐study time?
- How do you work with topics that are not covered in scheduled learning activities?

Topic B. Why do medical students choose to spend their self‐study time the way that they do?

- During self‐study, how do you pick which subjects to study?
- How do you prioritize your self‐study time if you have more to do than what you have time for?
  ○ What makes you do these priorities?
- What makes you choose to prepare for upcoming learning activities?
- What makes you choose to do follow‐up work after attending learning activities?
- What do you want to achieve with your self‐study?
- Do you wish you could organize your self‐study in a different way than what you actually do?
  ○ Why (not)?
- Has your self‐study behaviour changed during your time as a medical student? In what way?
  ○ Why?

Topic C. How do medical students plan for self‐study?

- At the beginning of a new semester, how do you plan your self‐study, in a full semester perspective?
- When do you start planning for next week's self‐study?
  ○ What are you taking into account when making these plans?

Topic D. What do medical students think of their organized time schedule?

- What do you think about the schedule for this study year?
- What do you wish was different?
- Do you think the amount of scheduled learning activities is adequate, too little or too much? Explain why.
- What does the ideal schedule look like to you?

Topic E. What learning resources do medical students use, and why?

- What learning resources do you use during self‐study?
- Why do you choose to use these?
- What do you think characterize a good learning resource?
- Learning resources you do not use—why do not you use these?

Final question

- Is there something you would like to add regarding how you use your self‐study time?

Interview data were thematically analysed using the phenomenologically inspired four‐step method Systematic Text Condensation (STC) [21]. In step 1, preliminary themes based on the entire data material were suggested and discussed among the authors. Ten preliminary themes emerged. In step 2, the themes were revised into four main code groups, each including 0–4 subgroups. Meaning units were identified and sorted into their relevant code/subgroup. In step 3 condensates—artificial quotations summarizing participants' statements—for each code group were composed. Step 4 involved making analytical texts based on the condensates from the previous step. These were revised by all authors collectively, which eventually resulted in three result categories. These categories and their content were checked against the entire data material and agreed on by all authors.

## 3 Results

### 3.1 Time Spent on Self‐Study

Average amount of time spent on scholastic activities was 42.1 h per week. Students spent slightly more time on self‐study than on scheduled learning activities (Table 1), accounting for 55% versus 45% of total scholastic time in average values, respectively. Thus, they allocated approximately 1.2 h self‐study per hour scheduled learning activity. More time was spent on preparations for upcoming learning activities, than on after‐class work (Table 2). Approximately 50% of self‐study time was done without specific relation to scheduled learning activities. Out of this, about 41% was exam related (preparing for exams, solving previous exam assignments). Other activities in this category included independent study unrelated to scheduled activities, student‐initiated colloquium groups and listening to scholastic podcasts, among other things.

**TABLE 1:** Time log data. Number of hours per week of scheduled learning activities, self‐study, nonscholastic activities and sleep. Values are given in median, first quartile (the lowest 25% of data being below this value) and third quartile (the lowest 75% of data being below this value).

Activity	Hours spent per week		
	Median	1^st^ quartile	3^rd^ quartile
Scheduled learning activities	18.8	16.3	20.8
Self‐study	24.3	15.3	27.5
Preparation	10.3	6.3	13.8
After‐class	0.0	0.0	2.3
Other	9.0	6.0	14.3
Nonscholastic activities	67.0	63.8	75.3
Sleep	58.3	53.0	61.8

**TABLE 2:** Time log data. Number of hours per week of scheduled learning activities and self‐study related to each learning activity. Values are given in median, with first quartile (the lowest 25% of data being below this value) and third quartile (the lowest 75% of data being below this value) being specified within brackets.

	Patient encounters Median (1^st^ quartile, 3^rd^ quartile)	Team‐based learning Median (1^st^ quartile, 3^rd^ quartile)	Problem‐based learning Median (1^st^ quartile, 3^rd^ quartile)
Scheduled learning activities (hours/week)	8.0 (6.0, 10.5)	8.5 (8.3, 9.3)	2.5 (2.3, 2.5)
Self‐study (hours/week)	3.0 (0.0, 6.0)	5.5 (3.0, 9.0)	0.5 (0.0, 2.0)
Preparation	2.0 (0.0, 5.3)	5.5 (2.8, 8.5)	0.5 (0.0, 2.0)
After‐class	0.0 (0.0, 2.0)	0.0 (0.0, 0.0)	0.0 (0.0, 0.0)
Ratio^a^	0.4	0.6	0.2

^a^ Ratio = time spent on self‐study/time spent on scheduled learning activities.

### 3.2 Qualitative Use of Self‐Study Time

Three main topics emerged from the analysis: (1) A change in study behaviour towards more preparation, (2) diverse learning activities yield different study behaviours, and (3) navigating learning resources for self‐study.

1 A change in study behaviour towards more preparation

Students reported spending most of their self‐study time on preparatory work for upcoming learning activities, rather than follow‐up work. This represented a change in study behaviour. In previous academic years, scheduled learning activities were mainly in the format of plenary lectures, which the students said they rarely had prepared for. Now, on the other hand, they were expected to be prepared and contribute actively in learning activities.

What has changed now, is that I have started to prepare [for the learning activities] (…). I usually did not prepare for lectures (…). (Student 2)

Being well prepared enabled them to discuss with fellow students, as well as to ask instructors in‐depth questions during the learning activities. Thus, the students experienced that preparation increased learning gains during the learning activities, which motivated them to prioritize preparatory work.

I just learn much less if I am not prepared. I end up sitting there having questions about fundamental concepts and I do not quite get how things are connected. However, if I have some knowledge about it before I get there, it goes more like ‘Yes, of course that is what happens, but why?’. It enables you to take the learning one step further. (Student 6)

Being a small group of students, they felt that their peers expected them to be prepared and to engage actively in the learning activities. Some expressed anxiousness about giving incorrect answers at the beginning of the year. However, this had changed over time, and at this point, they felt confident in both asking and responding to questions in class, including making mistakes.

Interviewer [I] How do you feel about that – to be addressed like that [in class]?

Respondent [R] In the beginning I felt very uncomfortable. But now, I feel prepared for it to happen, so it's fine. Yet, if I have not prepared very well and I get asked a question, it can be a little unpleasant if you do not know what you are talking about.

I A bit uncomfortable?

R Yes. But then again, the learning environment is so good it does not really matter if you come up with an incorrect answer. (Student 8)

2 Diverse learning activities yield different study behaviours

Students spent much of their self‐study time preparing for TBL and patient encounters, and less time preparing for PBL. TBL typically required in‐depth reading of a specific topic, and most students reported that topics of upcoming TBLs were of great importance for how they chose to spend their self‐study time.

I To what extent do the [TBL] seminars influence how you work in your self‐study time?

R They probably have a big impact. I think they play an important role in motivating me, as I know it is very good for me to be prepared. Because that will give me a sense of accomplishment when I attend the seminar. I find it rewarding to know that if I am prepared for tomorrow, then I will achieve a sense of accomplishment. That makes me really want to read up on it today. (Student 3)

Preparatory work prior to patient encounters was also highly prioritized. In addition to studying a specific topic, the preparations typically included reading up on the patient's medical history and practicing relevant clinical examination skills. The students claimed that patient encounters made studying more exciting. Knowing that they would have independent patient consultations encouraged them to prepare well. Some mentioned that ensuring patients had a positive experience with meeting students in clinics was an additional motivating factor. Being able to discuss with the supervising physician after the patient encounter was pointed out as another incentive to prepare.

It feels better when you come prepared. There is something about it; when you have a little bit of background knowledge it is easier to follow both the consultation and the discussion with the doctor afterwards. And then you are capable of actually discussing more, and that makes me feel that I benefit more, if I come prepared. (Student 4)

Less self‐study time was allocated to PBL preparations. One student said she previously had considered PBL as ‘a big thing’ that she used to feel obliged to prepare well for. Now, PBL did not put much stress on her anymore. It had been emphasized by the PBL facilitators that students could and should participate in PBL discussions even if they were unprepared. The PBL facilitators—being general practitioners—were described by students as highly suited for the role as facilitators, supporting the students in broad differential diagnostics and providing insight into how the health care system works at large.

He [the facilitator] said that he wanted us to do it [prepare for PBL] because we find it enjoyable. And that if you feel that you do not have time for it, he trusted us in the considerations we had made, deciding that we in fact did not have time for it. We should not burn ourselves out due to this. That contributed to lowering the threshold. And it makes it more fun to do the preparations (…). (Student 4)

3 Navigating learning resources for self‐study

Navigating the abundance of learning resources presented a dilemma for the students. They reported having access to a vast variety of both physical and digital learning resources, and they found it challenging to decide upon which to use.

And there are so many different resources; books, PowerPoints, YouTube‐videos, lectures, (…), so to determine which to use is challenging. (Student 1)

Books were used by several students, typically to get an overview of a subject. Online videos were also commonly utilized. PowerPoint slides and compendia derived from lectures held at the main campus were helpful in setting the bar and thus applied by students to direct their use of self‐study time.

I Do you use PowerPoint slides from lectures held in the main campus (…)?

R Yes, pretty often.

I What do you use it for?

R When I am preparing [for learning activities], to see how far I should delve into it. Where the bar is set at the main campus. If the PowerPoint slides are really good, or there is audio included, I will watch them and make notes.

(Student 6)

When the instructors recommended specific learning resources, the students often followed these suggestions.

## 4 Discussion

Students in the present longitudinally integrated curriculum spent on average 50% of their self‐study on preparations for, and follow‐up work after, scheduled learning activities. TBL, PBL and patient encounters had different impacts on student self‐study, both in quantity and quality. Students prioritized preparation over follow‐up work for all learning activities.

### 4.1 Amount of Self‐Study Time

Study participants spent on average 42.1 h per week on scholastic activities, whereof 55% (23 h) were self‐study. Investigations from other countries and settings have reported 26.5 [20], 21.6 [15], 9.8 [22] and 8.0 [23] hours of self‐study per week. However, variations in data collection methods complicate comparisons. Whereas some studies present time available for self‐study, others, like ours, base their numbers on actual time as reported by students. Other possible explanations for divergent findings include dissimilar curricular models and style courses, different pedagogies and differences among faculty perception of self‐study's significance. Moreover, different cultures and different education systems may approach this issue differently, which could elicit variations in study behaviour.

We found that students spent more time attending scheduled learning activities than preparing for and doing follow‐up work related to these activities (ratio 1.6:1). Although the optimal distribution between self‐study and instruction time is debated, noteworthy inquiry suggests a 1:2‐ratio for in‐class activities to self‐study [24]. Gijselars and Schmidt found that time spent self‐studying was more crucial for students' exam achievements, than time spent on instructional activities [14]. They claim that students have a saturation point for time they are willing to spend on education (in their study 37 h per week). Therefore, they argue that instruction time should be limited, to allow for sufficient number of hours available for self‐study. Schmidt suggested that student learning depends on time available for independent and active learning. Hence, if a curriculum employs activities that do not facilitate these learning processes, learning will actually be restrained [15].

### 4.2 Content of Self‐Study in Relation to Scheduled Activities

The interview data suggest that different learning activities motivate different learning behaviours. TBL played a major role in students' acquisition of theoretical knowledge; patient encounters encouraged them to practice clinical skills, whereas PBL to a lesser extent influenced their self‐study. Nevertheless, most students found PBL useful as it prompted broad differential diagnostic reasoning. Thus, this study indicates that the three learning activity types complement each other.

Students' assertions that self‐study related to TBL and patient encounters exceed self‐study related to PBL are supported by time log findings when considering absolute number of hours. Estimation of ratios for scheduled hours to self‐study hours per activity, however, shows that these differences partly even out. There are several possible explanations for TBL and patient encounters encouraging more self‐study than PBL. Students may perceive the learning potential as higher in TBL and patient encounters; thus, they will benefit more from preparing for these activities. This could be due to TBL being a more instructor‐led activity, implying that the session content is carefully selected and reviewed by an instructor. Encountering an overwhelming syllabus this can be a welcome help for students, also in navigating topics relevant for their exams. Furthermore, TBL includes assessments both in an individual format and in group format. Despite these assessments being strictly formative in our curriculum, the awareness that you will be tested may promote self‐study motivation. In PBL sessions on the other hand, a facilitator is present, but the progress and the academic content are more student led. Patient encounters most often comprise situations in which students' skills and knowledge are being tested. Students possibly feel a greater sense of responsibility for being prepared for TBL and patient encounters, both towards their peers, patients and instructors—perhaps because of the more ‘confronting’ format of TBL and patient encounters, as outlined above.

Students described an alteration of self‐study allocation from their two first study years to years 3 and 4. During years 1 and 2, they spent less time preparing for learning activities, whereas in the later years, preparation was highly prioritized. As this study was not designed to establish causality, we can only hypothesize about reasons for this behavioural change. One suggestion could be that students have gained more experience and have matured as learners when they reach years 3 and 4, resulting in abilities to choose more beneficial study strategies. Alternatively, the study behaviour change could be related to the shift in types of learning activities offered to the students. Change in study behaviour towards prioritizing preparation when commencing active learning pedagogies has been described in other curricula [25].

Preparing for scheduled learning activities is in line with the pedagogic theory of active learning sessions [18, 26]. Both theoretically and empirically, the retrieval of previously acquired knowledge is important for the learning process. Valuable in‐class time does not need to be spent on conveying knowledge that students are perfectly capable of obtaining independently, but rather on assisting students' deeper understandings and development of reflective and critical thinking [24, 27]. Our findings align with the notion that applying active learning methods encourages preparation.

### 4.3 Other Determinants of Self‐Study Behaviour

In addition to scheduled learning activities, content of lectures given at the main campus stood out as determinative of students' self‐study priorities. The latter seemed to be closely related to exam preparations, as these instructors created the exam papers. Previous research has identified various curricular components that influence self‐study including course objectives, discussion in tutorial groups, lectures, content tested, tutors and suggested literature [28, 29]. Notably, our time log findings reveal that 50% of student self‐study were not related to scheduled learning activities, but rather, for example, exam preparations and student‐initiated colloquium groups. This illustrates that despite the application of active learning methods aiming to promote self‐study, other factors also affect how students choose to spend their self‐study time.

Assessment as a driver for learning has been widely addressed in medical education [30]. Our study illustrates the added stress students experience when exam setters differ from their instructors. This emphasizes the importance of alignment between (1) intended learning outcomes, (2) teaching/learning activities and (3) assessment tasks—a concept known as *constructive alignment* [31]. In our study, students expressed concern about a lack of alignment between learning activities and assessments. This study was not designed to evaluate this (lack of) alignment. However, further studies can investigate factual alignment between instruction and exam content. The notion of constructive alignment can be further extended to include learning resources. Offering students learning resources that align with learning outcomes, learning activities and assessments may fill their apparent need for guidance in this regard. A thorough alignment between these elements could also provide the opportunity for using learning outcomes and learning resources as indicators for setting the bar, instead of lecture slides and previous exam questions.

### 4.4 Strengths and Limitations

Strengths of this study include the mixed‐methods approach, allowing a qualitative elaboration of students' reasoning and motivation regarding self‐study, as well as robust quantitative data on actual time allocation. This enabled both a triangulation of data and a deeper insight into self‐study time use than previous studies have offered. Furthermore, the daily self‐report time logs reduced the risk of recall bias. As the study's context was a decentralized longitudinal integrated curriculum, findings may be particularly relevant for programmes organized similarly.

Limitations of this study include the small number of interviewees, who all affiliated to the same curriculum, which may limit the transferability of the results to other curricula. This methodological decision was made due to the intention of conducting an in‐depth analysis of the current setting. A variant of the Hawthorne effect, which entails participants adjusting their behaviour because they know they are being observed, could potentially have affected the time log results. Increasing the number of participants for this part of the study was a remedial intervention to minimize this effect. Moreover, time log registrations occurred in specified weeks and thus may not be representative for other parts of the semester.

## 5 Conclusions

Students spent on average 23 h per week on self‐study, amounting to 1.2 h of self‐study per hour scheduled learning activity. In keeping with pedagogic intentions of the applied learning methods, large proportions of the self‐study time were dedicated to preparation for upcoming activities. Different learning activities promoted various self‐study behaviours; TBL motivated acquisition of theoretical knowledge and patient encounters motivated practice of clinical skills. However, approximately 50% of student self‐study time was unrelated to their scheduled learning activities, of which exam preparations made up a considerable fraction. Further research should explore how self‐study behaviour is affected by quantity and content of scheduled learning activities.

## Author Contributions

**Ida Sara Johnsen:** conceptualization, investigation, methodology, formal analysis, writing – original draft, writing – review and editing. **Vidar Gynnild:** conceptualization, methodology, formal analysis, writing – review and editing. **Børge Lillebo:** conceptualization, methodology, formal analysis, writing – review and editing.

## Ethics Statement

Ethical approval was obtained by the Norwegian Centre for Research Data (NSD number 450319).

## Consent

Informed written consent was obtained from all individual participants included in the study.

## Conflicts of Interest

The authors declare no conflicts of interest.

## Data Availability

The data that support the findings of this study are available from the corresponding author upon request.
